# Biological Activity of Essential Oils

**DOI:** 10.3390/molecules25030678

**Published:** 2020-02-05

**Authors:** Francesca Mancianti, Valentina Virginia Ebani

**Affiliations:** 1 Dipartimento di Scienze Veterinarie, Università di Pisa, Viale delle Piagge, 2 I 56124 Pisa, Italy; valentina.virginia.ebani@unipi.it; 2 Centro Interdipartimentale di Ricerca Nutraceutica e Alimentazione per la Salute “Nutrafood”, Università di Pisa, Via del Borghetto, 80 I 56124 Pisa, Italy

Essential oils (EOs) have for a long time been recognized to possess several different biological activities. Several among these secondary plant metabolites exhibit marked antimicrobial effects that have made their use as an antiseptic and/or preservative in food well known, since the ancient times.

In this Special Issue, several oils extracted from plants growing in different environments and frequently employed in ethnomedicine were investigated in 30 original research and two review papers. This issue encompasses both in vivo and in vitro studies.

Some novel approaches are reported in both selecting and delivering them. In particular, machine learning algorithms were applied to analyze the chemical composition of the EOs to understand how it can be related to the anti-biofilm potencies [1]. On the other hand, the use of biomaterials with properties for improving antimicrobial activity is up to date, and coating hydroxyapatite with peppermint EO would significantly enhance its antibacterial effect [2].

The composition of the EOs within the same botanical species is influenced by various parameters such as time of harvest, mode of extraction, and conservation [3]. Therefore, in the present issue, a strong correlation between the chemical composition and environmental parameters (geographical position, bioclimate, and nature of soils) of individual oil samples extracted from aerial parts of *Eryngium campestre* was reported [4].

The varying composition of EOs makes it difficult to compare their antimicrobial activities, however, there is a need to understand the antimicrobial effects of EOs to gain insights into their application potential [5]. In this framework, a critical, excellent review of the antimicrobial activity of EOs is provided by Winska et al. [6], with the view to promote further investigations into targeting EOs and microorganisms.

Based on this information, in this Special Issue, most EOs have been screened for their in vitro antibacterial/antifungal activity, and in some selected papers, the micro-organisms were chosen from the perspective of a practical application to face different situations in the field. *Clausena lausium* EO showed potent activity against *Candida* yeasts, corroborating the efficacy of these traditional remedies in Chinese folk medicine [7], while *Cymbopogon flexuosum, Litsea cubeba*, and *Citrus bergamia* EOs appeared active against *Saprolegnia parasitica* [8]. However, attention should be given to in vivo administration of such products, being that some EOs have been proven to exert strong antimicrobial effects in animal practice where they would show toxic effects damaging the liver, kidney or gastrointestinal tissues [9].

Although the use of EOs as antimicrobials has been reported to not induce relevant changes in the sensitivity to antibacterial drugs [10], a novel insight into the mechanisms of action and *Staphylococcus aureus* resistance against active individual constituents such as carvacrol, citral, and (+)-limonene oxide was provided by Berdejo et al. [11]. Selected microorganisms, exposed to sub-inhibitory doses of such compounds showed a higher tolerance to lethal treatments by individual constituents or heat. This investigation suggests the need for further studies aiming to evaluate whether the emergence of resistant strains in the presence of EOs and their main components is a general phenomenon.

Antiparasitic activity has also been assessed in vitro by indigenous essential oils against flagellate protozoa. In detail, *Endlicheria bracteolata* EO scored active against both culture amastigote and promastigote stages of *Leishmania amazonensis* [12], whilst some Vietnamese EOs were checked versus *Trypanosoma brucei brucei*. Among them, *Curcuma longa* was effective and, in particular its main component, curlone, appeared as a promising anti-trypanosomal candidate [13].

EOs have shown remarkable healing effects in wounds both in vivo and in vitro models [14] and a contributor to the potential of wound healing by *Eugenia dysenterica* EO has also been reported [15]. Although EOs have antiviral actions against animal/human viruses [16,17], Vuko et al. [18] reported a further study on the anti-phytoviral activity of *Micromeria croatica* EO in inhibiting satellite RNA Associated Cucumber Mosaic Virus Infection.

In a green approach to crop protection, EOs from *Coriandrum sativum* [19] and *Solidago canadensis* appeared active against several phytopathogenic bacteria and fungi [20] as well as geranium and rosewood EOs versus *Fusarium graminearum* [21].

EOs can also act as allelochemicals, in order to defend the plants against predators and other competing plants. *Xanthium strumarium* leaves EO was successfully tested as a green bioherbicide [22], while the phytotoxic activity of *Heracleum mantegazzianum*, an alien botanical species, was assessed on monocot and dicot plants species [23].

Among other less investigated biological effects, there is an anticancer potential of some EOs, checked in vitro and/or in vivo. EO obtained from Gannanzao orange showed a significant inhibition in the proliferation and migration of hepatoma and colorectal cancer cells [24]. The latter were sensitive to EO from *Origanum onites,* which was also able to inhibit in vivo the growth of syngeneic CT26 colon tumors when used for prophylactic oral administration in a murine experimental model [25]. Of interest also is the study by de Lima et al. [26] regarding the in vivo anticancer effect of EO from the leaves of *Croton matourensis*, a medicinal plant from Brazil.

Tea seed EO showed a marked anti-obesity effect in a murine model, which was able to prevent obesity, reduce physical fatigue, and improve exercise performance, suggesting a possible use for menopause-related metabolic syndromes [27].

In a perspective to investigate the therapeutic potential of organosulfur compounds for various disorders with immune and/or inflammatory mechanisms, garlic EO and some organosulfur compounds could serve as biological response modifiers by augmenting phagocyte functions. They were in fact able to activate neutrophil functional activity, suggesting that neutrophil stimulation by organosulfur components from *Allium* spp. might enhance resistance to infection [28].

Finally, thee antispasmodic effects of EOs from 39 botanical species were reviewed by Codrura Heghes et al, [29], indicating such compounds as promising alternatives to conventional antispasmodic drugs.
